# History of a Fatal Case of Variola Post Variolam Vaccinam

**Published:** 1820-07

**Authors:** Edward Harrison


FOR THE LONDON MEDICAL AND PHYSICAL JOURNAL.

### *History of a Fatal Case of Variola post Variolam Vaccinam.*

By Edward Harrison, M.D. F.R.A.S. ED. &c.

MASTER C. C. aged sixteen years, of a sanguine temperament and good habit of body, complained first, on Thursday, March 16th, of a sore throat, which has been increasing ever since. Becoming feverish the following morning, his mother gave him three grains of calomel and some senna tea. The fever rose higher on Saturday, in the course of which day the tea procured several motions. On Sunday, being more feverish and somewhat delirious, the medical attendant purged him, and took away twelve ounces of blood, which showed nothing extraordinary. Some spots were first observed on his arms the next morning: these, on Tuesday, had spread over his face, arms, legs, and body; from which time they continued to increase in number, and the fever became more violent. On being consulted this forenoon, March 22d, 1820, I find the face swollen, especially the cheeks and upper lip. The face, neck, legs, and thighs, are closely covered with a hard papular eruption, which, in a few of the spots, especially about the neck, contain purulent matter, or fluid of a milky colour. They are distinct on the body, but confluent about the nose, mouth, lower jaw, and neck. They are very numerous on the extremities and upper part of the face, though they may perhaps be more strictly denominated coherent than confluent. They are chiefly hard, round, and tubercular or warty, and without inflamed bases. These, according to the information given by the mother and attendants, have, from the beginning, been neither purulent nor vesicular. Most of them are flattened at the top, and covered with a dark-red or purplish scab or incrustation; few, if any, of them are depressed in the middle. They are quite dry, except a very small number, dispersed over the arms and lower limbs, which contain purulent matter: these bear a strong resemblance to the pustules in small-pox. A rubeolous rash, or erythematous efflorescence, and petechiæ, can be distinguished in the skin of the arms, legs, and thighs. He complains of great pain in the small of the back, and, on pressure, the epigastrium is very tender. There has been no sickness. The eyes discharge copiously a thick, tenacious, and whitish mucus, which has nearly glued-up the eye-lids. The skin feels hot and dry. There is a great deal of fever, and he is delirious, with a distressing head-ach. Has had no rigors nor chilliness. The throat is painful, and he swallows with difficulty. He gargles frequently, and gets rid of viscid mucus in considerable quantities. Tongue moist. Pulse 110, quick, and moderately strong. Belly rather open. The small-pox has been lately prevalent in the neighbourhood of his father's house. He was vaccinated in the right arm when only a month old, by Dr. Clarke, the late teacher of midwifery. The scar is very apparent, and the doctor reported him to have gone through a satisfactory form of the disease.

Ordered to drink red wine plentifully, mixed with lemon-juice and water, together with nutritive diet.

℞. Pulv. cinchon. lancifol. gr. xv; T. cinchon. c. ʒij; sod. subcarbonat. ℈ſs; sp. æther. composit. ʒſs; aqu. ℥i. Haustus 4tis horis sumend. superbibat succi limon. cochl. iij parvul.

℞. Hydr. submuriat. gr. ij; P. antimonial, gr. i. Confect. fiat bolus h. s. sumendus.

℞. Acid. muriatic, ʒj; decoct. hordei ℔j; syr. ℥j. Sumatur quotidie ad instar potus.

*March 23d*, 11 *a.m.*—Has had a good night. The fever and delirium continue. Throat discharges considerably, but he swallows with greater ease. The lips and cheeks are more swollen. Eruption is fuller, and more raised about the face. Where it is quite distinct, they are round elevated pustules, containing a purulent fluid. Those on the arms and legs are chiefly hard scabs, and may be called horny. The livid hue is considerably increased. Petechiæ this day much more numerous upon the extremities. On the outside of the fore-arm there is a gangrenous spot, five or six inches long and several inches broad. There are several smaller ones in different parts of the body and extremities.

Is ordered to take a pint of red port, and four ounces of lemon-juice mixed with it, in the twenty-four hours. Pulse 98; bowels regular. All the medicines agree well. The medicines to be continued, with the addition of a scruple of pulv. cinchonæ to each draught.

*Seven, p.m.*—At five this afternoon, the muriatic acid was discontinued, and the medicines changed for the following:

℞. Decocti cinchon. lancifol. ℥j; T. cinchon. c. ʒij; acid. sulphuric. dilut. min. xij.

The purple hue of the eruptions, the petechiæ, and the gangrenous suffusions, are visibly better since the morning.

*March 24th.*—The face is less tumid, and the pustules upon it have every character of small-pox. The peculiar variolous smell is very strong. The fever, though rather less, is considerable; but he is less delirious. Ptyalism abated. Since yesterday he has frequently spit up sloughs, pieces of membrane, and little round bodies; which are always mixed with a ropy fluid. He swallows with much more ease. Pulse in the evening 120.

*25th.*—The face is less swollen, and the pustules are all encrusted. I regret that they were not opened the first day, to examine the enclosed fluid: when the attempt was made to-day, no moisture could be obtained. I can however venture to assert, from careful and minute attention to the state of them on every part of the body, that, in all, the complexion of the pustules was turbid. The ankles have begun to swell. Many of the petechiæ have disappeared, and the suffusions are reduced in extent. Smell continues. Delirium removed. Morning, pulse 100; evening, 110.

*26th.*—Ptyalism and swelling of the face are gone. The hands and arms are much swelled and painful, as well as the feet, ankles, and knees. The eruptions are quite dry and scabbed every-where. The gangrenous suffusions are much contracted, and brighter. The petechiæ and erythema have disappeared. Peculiar smell still perceptible. Pulse 95, morning; 102, evening.

Habeat omn. nocte tinctur. opii min. xv. et liquor. antimon. tartar. min. x.

28*th*.—The elbows, wrists, and hands, are swelled and painful, as well as the knees, ankles, and feet. The gangrenous appearance of the left arm is considerably diminished in size, and much fainter. Several of the scabs have already separated, leaving little depression or discoloration of the skin. The variolous fetor has entirely ceased. Skin quite cool. Pulse 92 this forenoon.

Contin. haustus, sextis horis solummodo.

30*th*.—Yesterday afternoon he was seized with a smart accession of fever, which continued the greater part of the night. This morning his mother first perceived a numerous crop of limpid vesicles, or phlyctenæ, upon his shoulders and trunk, varying from the size of millet-seeds to that of a split-pea. After remaining an hour or two, they disappeared again; and have through the day been succeeded by similar ones. The shoulders and upper part of the body are become quite free from them; they are now confined chiefly to the legs and feet. The swelling of the extremities, though not gone, is lessened. Pulse 97, 7 *p.m.*

*April* 1*st*.—Several successive crops of small, round, and plump vesicles, like those described yesterday, and containing a colourless fluid, have appeared in the course of the day, chiefly on the body. The knees, ankles, feet, and elbows, are less swelled; they admit of being moved with more freedom and ease. The wrists and hands remain painful and swelled. Had but little increase of fever last night. Pulse 95, rather full and quick, 3 *p.m.*

2*d*.—The vesicles are numerous on the face and lower part of the belly; they have never become turbid. The lower extremities have ceased to give much pain, and admit of his moving them. The left hand and wrist little swollen; the wrist of the right hand appears red, swelled, and very painful: the hand is quite well. He has used the following with great benefit since the 28th March:

℞. Decoct. cinchon. ℥vj; liquor. ammon. acet. ℥ij; T. opii, ʒij. M. f. lotio, cujus, mediocriter calefacti, appl. paululum partibus affectis frequenter, linteo bene madef.

4*th*.—The eruptions disappeared entirely yesterday afternoon. He still complains of his wrists, knees, and ankles; all of which are red and inflamed.

Let a linseed poultice be applied to the right hand and wrist; the lotion to the other parts. Capiat noct. gr. ij; hydr. submur. cum pulv. antimon. gr. j. Cras mane h. purgant.

*5th.*—This morning an attack of hemoptysis suddenly took place. He discharged more than half a pint of florid and very tenacious blood. It returned again in the afternoon, and he was immediately suffocated with the hemorrhage.

Dissection of the body was performed on the day after his death. On the left side were found numerous adhesions of the pleura costalis and pulmonalis. The left lung was much condensed; several portions of it sunk in water. Tubercles were found in different stages; and some of them had suppurated. The ramifications of the bronchiæ were in many places lined with a false membrane; they were full of a bloody fluid, and contained numerous tenacious ropy substances, probably from the red particles of the blood being entangled in the fibrin. The right lung was hepatized; it adhered strongly to the diaphragm over the liver: in other respects it was but little affected. The jejunum was unusually vascular in many parts, and was there greatly distended: it contained an unusual quantity of mucus. The coats of the ilium were lined with thick, brown, and adherent mucus. The blood-vessels were greatly distended, and contained very dark blood. The gall-bladder was nearly empty: it contained a fluid which resembled the discharge in virulent gonorrhœa. The abscess in the left wrist was in contact with the radius, which was deprived of its periosteum. The abscesses in the other wrist and in the knee were not examined.

***Bulstrode-street; May 9th,*** **1820.**

